# A New Intervention Procedure for Improving Classroom Behavior of Neglected Children: Say Do Say Correspondence Training

**DOI:** 10.3390/ijerph16152688

**Published:** 2019-07-27

**Authors:** María J Pino, Javier Herruzo, Carlos Herruzo

**Affiliations:** Department of Psychology, Faculty of Education, University of Cordoba, 14071 Córdoba, Spain

**Keywords:** neglect, intervention, say-do-say correspondence training

## Abstract

Although neglect is the most common form of child maltreatment, a review of the literature since 1980 reveals a lack of controlled child neglect intervention programs. The aim of this study is to assess a new intervention program to improve the classroom behavior of children exposed to neglect only, by reducing disruptive conduct and promoting adaptive conduct. Two matched groups were selected with children of the same ages, sex, and social class (cultural and economic level) and with mothers of similar ages. The experimental group comprised of five children suffering from neglect and no other type of maltreatment. The control group had five children not abused or neglected. All the children were in the same class at school. The percentage of time per session that each child spent engaged in disruptive behavior was measured (baseline) and was found significantly higher among neglected children. Say-Do-Say Correspondence Training was applied with the neglected children and a rapid, significant reduction in their disruptive behavior was observed (and statistically confirmed), bringing such behavior down to the level of the control (i.e., non-neglected) children. These results were maintained when the intervention was halted. We concluded that the adaptive and classroom behavior of neglected children can be improved with this non-intrusive intervention.

## 1. Introduction

Child neglect is a highly prevalent form of child maltreatment and has been repeatedly identified in many studies [1,2,3,4,5,6,7]. It is defined as the failure of a parent or another person with responsibility for a child to provide the food, clothing, shelter, medical care, or supervision required to ensure that the child’s health, safety, and well-being are not harmed [4,6]. It is estimated, for example, that approximately 8 out of 1000 children in the USA and approximately 6 out of 10,000 children in Spain are neglected. However, figures vary considerably depending on the region, the source of information, the age range considered, and the definition and instruments used [3,8]. Even so, despite its high prevalence, neglect has received relatively little attention [3,7,8,9,10].

Despite the difficulty of studying pure typologies, neglect has been associated with negative social, behavioral, and cognitive consequences [11,12,13,14]. During childhood, slow development problems have been reported, with most behavioral areas being affected (e.g., [15]). Issues reported include attention deficit and cognitive problems, difficulties with communication and expressive skills, lower academic achievement, altered emotional behavior, fewer social skills like empathy and interpersonal relationships, and more difficulties in social interaction, with distorted patterns of interaction with carers and peers [16,17]. All this can alter children’s brain development and physiology, impact learning and behavior, and increase their risk of poor physical and mental health [18,19].

According to numerous studies, children who grow up in neglectful or abusive homes suffer from impairments to their basic trust, self-esteem, and ability to form and maintain relationships, and to their affective development (see reference [20]). Later, as adults, they are also prone to serious personality disorders and other psychopathologies [21]. Their academic performance tends to be poor and their cognitive development delayed. They run a higher risk of severe behavior problems, from non-compliance and temper tantrums through delinquency, violence, and other forms of anti-social behavior [17,22,23,24,25]. In contrast to physically abused children, neglected children have more serious cognitive deficits and socialization problems, and appear to display more internalizing behavior patterns as opposed to externalizing patterns [12,26,27,28].

Neglected children have been found by both parents and teachers to display more internalizing behavior problems than other children [29,30]. Kotch et al. [31] found that children who have been abused or neglected are at high risk of displaying externalizing behavior problems, and this may later snowball into aggressive and criminal behavior [32], especially if it occurs before the age of five [31]. The severity of physical neglect, particularly during the preschool period, has been associated with internalizing symptomatology and withdrawn behavior [33]. Woodruff and Lee [34] found that in neglected children with internalizing behavior problems, those problems tended to grow worse. As Oh and Song [12] recently found, childhood abuse and neglect have a significant, direct negative effect on school adjustment, the impact of neglect being bigger than that of abuse.

As Lemkin, Kistin, Cabral, Aschengrau, and Bair-Merritt [23] pointed out, supporting educational success provides a great opportunity to help maltreated young people make positive progress since educational achievement and health are positively correlated. This last aspect emphasizes the importance of intervening as soon as possible to prevent behavior problems from developing or worsening. As some reviews of the literature since 1976 [2,3,35] indicate, however, there is a considerable lack of controlled child neglect intervention programs, including programs focusing on victims of childhood neglect and (or) their caregivers. The conclusion is that the effectiveness of treatment for children exposed to neglect only (without the co-occurrence of abuse, as is often the case in marginal areas, where caregivers lack the skills to effectively care for their children and, as a result, neglect them but do not physically abuse them) cannot be determined from the existing literature.

Another key challenge for research in the area of child neglect is the obtention of samples in which exposure to neglect and exposure to abuse do not overlap [2,3,36].

The main results obtained have come from play behavior. Thus, Fantuzzo et al. [37] used play therapy, which involved pairing a resilient peer with a target child for 15 play sessions, supervised by an adult assistant, to improve the withdrawn child’s social interaction and to increase positive, interactive peer play. Two months after the treatment, improvements were observed in social interaction among the children treated, with fewer internalizing and externalizing behavior problems.

Udwin’s [38] intervention consisted of group training sessions in imaginative play, conducted over five weeks. Preschool children in the experimental group were found to have improved in terms of imagination, cooperation, and interaction with peers, and their aggressive play had decreased in comparison with the control subjects. Play therapy programs have, therefore, had positive results and it is important to replicate them in order to determine which specific aspects of such programs might help children exposed to neglect, although this falls outside the scope of this work.

Although improvements have been demonstrated in play behaviors, no intervention has yet been conducted to improve classroom behaviors [39]. It has been suggested in the literature [23,30,31,32,33] that supporting educational success provides a great opportunity to help maltreated young people make positive progress and halt the usually worsening evolution of these children, thus, the aim of this study is to assess a new intervention program to improve the classroom behavior of children exposed to neglect only, reducing disruptive conduct and promoting adaptive conduct. There is one behavioral modification procedure that has been demonstrably efficient in improving classroom behavior among delayed and non-delayed children and reducing disruptive behaviors in educational settings. This procedure is known as correspondence (saying-doing) training [40]. It is a strategy used in applied research to increase self-management skills in young children. Its aim is to teach a child to do what he or she has said they will do. This procedure, first used by Risley and Hart [41], has been employed both to promote adaptive behaviors and reduce disruptive behaviors [42], addressing such issues as on-task behavior [43,44,45], social behaviors [46,47,48], compliance [49,50], self-care skills [51], and verbally mediated responses [52]. However, it has not yet been used with neglected children in an applied context.

One particular type of correspondence training procedure, known as say-do-say or say-do-report correspondence training [53,54], could swiftly reduce problematic behaviors without disrupting daily classroom activities or being too intrusive. The say-do-report procedure has been seen to have the highest level of empirical support [36,53,55] of all correspondence training procedures. Herruzo, Luciano, and Pino [56] applied this procedure to eliminate disruptive behaviors in groups during free time activities.

In this study, a group of 10 children between the ages of 9 and 12 verbalized (“say”) that they were not going to behave inappropriately (they specified the behaviors and the situation) and were then encouraged to carry out (“do”) what they had verbalized. A series of questions was then formulated aimed at fostering the child’s understanding of what he had said, what he had then done, and the relationship between the two (the correspondence between saying and doing). As Herruzo and Pino [57] demonstrated in an experimental study, neglected children did acquire the say-do correspondence repertoire for simple behaviors, but a significantly greater number of trials were required than for other more resilient peers. However, implementing this technique in the classroom with younger children could be more problematic, since individual attention is required. Specifically, the time needed to learn the correspondence repertoire (several attempts at least) could be an issue, especially for children with attention deficit disorders. As Pino and Herruzo [20] suggested, it might, therefore, be better to carry out specific training in this repertoire outside the classroom on a more individual basis, thus that the correspondence repertoire could be learned and generalized to other behaviors in a session lasting between 30 and 60 min. Thus, once the repertoire has been acquired and generalized at an individual level, the procedure could then be implemented by the teacher in the classroom, since it would simply be a question of generalizing the pre-acquired repertoire to new behaviors and contexts.

The aim of this study is, therefore, to improve classroom behaviors of neglected children using say-do-say correspondence training. First, the experimenter will apply the procedure outside of the classroom until the children manage to acquire and generalize the say-do correspondence to other, new, simple behaviors. Once the children have generalized the correspondence repertoire, it can then be applied in the classroom by the teacher with behaviors that would lead to an improvement in the child’s adjustment and school performance (reducing disruptive behaviors and improving adaptive behaviors). It is also hoped that the teacher him/herself will apply this procedure in the classroom, thus making its application more economical and less intrusive. The main hypothesis is that if neglected children learn say-do correspondence with a simple behavior outside of the classroom and generalize it to other, new, simple behaviors when the teacher applies the correspondence training in the classroom, a more rapid improvement will be observed in the children’s behavior, making the intervention less intrusive. Changes in classroom behavior are only expected to occur in those children who received the intervention, not in the control group of peers.

## 2. Materials and Methods

### 2.1. Participants

In the second year of kindergarten, 10 of the 18 children (aged between 4 and 5; average = 4 years 4 months) at a school in a city in Andalusia (Spain) took part in the study. Of the intervention group, 5 of them suffered from the neglect subtype of maltreatment and all 5 were included in the study. The kindergarten was in a poor neighborhood of the city and Social Services were able to provide a lot of information about all the pupils at the school. Neglect was diagnosed using the Abuse Indicators designed by Arruabarrena, De Paúl, and Torres [58] (see Appendix A) and a great deal of data recorded during very frequent Social Services visits to monitor the children’s families. The other 5 children (the control group) were recruited from the other 13 children in the class group, who had not been neglected or exposed to any other form of maltreatment. These 5 were randomly selected from the 13 to make up a group analogous to the intervention group in terms of age, sex, social class (cultural and economic level), and their mother’s age. Their level of development was measured using the Inventory for Client and Agency Planning, ICAP [59]. The development of the 5 subjects suffering from neglect was on average 12 months behind that expected level for their age, whereas for the 5 subjects in the control group it was 7 months ahead of that expected for their age. All information was provided by local social workers, the children’s teachers, and the authors’ own assessment. The control group made up of non-neglected children was included in the study in order to improve internal validity and to give the researchers a point of reference for normal behavior inside the classroom in this specific context.

All subjects’ parents/caregivers gave their informed consent for inclusion before they participated in the study. The study was conducted in accordance with the Declaration of Helsinki, and the protocol was approved by the Ethics Committee of Andalusian Govern Research Group HUM775.

### 2.2. Measurements, Settings, and Instruments

The problematic behaviors displayed in the classroom by the target children were as follows:

Standing up: Being out of their chair when an activity required them to be seated.

Absent: Talking to other children or looking in a different direction from the focus of attention of the activity that was being carried out. Absence was also taken to mean when the child was absorbed by an individual activity (for example looking at or playing with a small toy they had in their pocket or turning around in their chair and playing with school material behind them).

Disruptive: Fighting with, shouting at, or disturbing other pupils.

All 3 types of behavior were observed during 3 activities carried out in the kindergarten classroom in the morning for 2 hours (the duration of each session) before playtime. The first activity was Assembly, in which dialogue was conducted between the teacher and the children, on various topics. A Psychomotor task was then carried out, in which the teacher gave instructions that the children had to follow. Finally, there was a Worksheet activity (taken from a Kindergarten textbook).

The intervention was carried out in different rooms at the aforementioned kindergarten: The children’s usual classroom, where the teacher implemented the intervention regarding inappropriate behavior; and outside the classroom, in an office area, and some adjacent rooms that were familiar to the children.

In this study, a recording device was also used to record the interactions in the office, and paper to write down these entries. Different toys, surprise envelopes, and sweets were used as reinforcers.

### 2.3. Design

The study was divided into 3 stages, sequenced as follows. (I) Baseline study of disruptive behaviors in the classroom. (II) Intervention outside the classroom: S-D-S Correspondence Training outside the classroom with a simple behavior (B1: Touching an abstract picture on a panel) and generalization to another two behaviors (B2: Putting something inside a box, and B3: Picking an object up from the floor). (III) Intervention inside the classroom: Say-Do-Say (S-D-S) correspondence training for several behaviors carried out by the teacher and partial withdrawal from the intervention.

The intervention inside the classroom was assessed using a single case experimental design for 10 participants (divided into 2 groups) with 3 stages A (I) B1 (II) B2 (III) serving as a group design with a control group and pre-test or baseline measurements and post-test or intervention measurements. The intervention was carried out with the experimental (intervention) group (5 children suffering from neglect). The control group was made up of 5 non-neglected children similar to the first group in terms of age, sex, social class, and their mothers’ ages.

The dependent variable was the percentage of time each child spent behaving inappropriately, engaging in any of the 3 inappropriate behaviors defined previously (standing up, being absent during the task, and being disruptive) per session.

Intervention outside the classroom to acquire and generalize the say-do correspondence repertoire constituted an experiment in itself and followed the stages described in Herruzo and Pino [57]. For the sake of concision, therefore, please see the open access references listed at the end of this article. This intervention took place between sessions 10 (the last baseline session) and 11 (the first session of intervention inside the classroom). This enabled the subjects to acquire the say-do correspondence for Behavior B1 (touching a picture in a panel) and generalize it to the two new behaviors (B2: Putting something inside a box, and B3: Picking an object up from the floor).

### 2.4. Procedure

#### 2.4.1. Baseline Disruptive Behaviors in the Classroom

For 10 sessions, 3 trained observers recorded the occurrence of the behaviors “Standing up”, “Being Absent”, and “Being Disruptive”, described in the section on behaviors. The recording method used was systematic sampling at 25 s intervals. In other words, every 25 s, a mobile device indicated that it was the time for observation and recording, which was done for the next 5 seconds. During these 5 seconds, the observers checked off what the children were doing in the relevant box (standing up, disruptive, absent). The 3 observers acted as follows: Observer 1 recorded the experimental group children every day, Observer 2 recorded the control group children every day; and Observer 3 recorded both groups alternately: One day the experimental group and the following day the control group. In the end, the percentage of time spent engaged in each behavior was estimated based on the number of intervals in which the occurrence of these behaviors had been recorded. For 2 days after the 10 pre-test sessions, no observational measurements were recorded inside the classroom and the children from the experimental group were taken out of class one by one to receive “intervention outside the classroom”.

#### 2.4.2. Intervention outside the Classroom (Conducted by the Authors)

This intervention was only conducted with the children from the experimental group. As has been described in the Design section, S-D-S Correspondence training was applied outside the classroom in spaces that were familiar to the children. The procedure was very like that applied inside the classroom, which will be described in the next section, but here it was conducted individually. It was applied in short sessions of 10 to 15 minutes and continued until each child achieved the correspondence repertoire in B1 and generalized it to B2 and B3. These sessions took place over 2 days.

#### 2.4.3. Intervention inside the Classroom (Conducted by the Teacher)Say-Do-Say Correspondence Training for Several Behaviors, Conducted by the Teacher, and Partial Withdrawal from the Intervention

One day before the intervention began, the teacher was shown the procedure and several trial runs were carried out (as roleplays). Since the session was divided into the 3 activities indicated above (assembly, psychomotor functions, and worksheet activity), the S-D correspondence interactions were programmed with the children in relation to these actions. The psychomotor tasks usually took place during the assembly, when 4 predefined interactions were set: Assembly (part one), psychomotor tasks, Assembly (part two), and worksheet activity. Thus, when the assembly was about to start, the teacher got the 5 experimental children into a little group, and the following verbal interaction took place (see Table 1).

These questions were then repeated with the other children. From the 3rd day on (session 13), however, the question was asked to one of the children, and the others were then immediately asked “And what about you?”, thus the verbal interaction with the children was quicker. When this verbal interaction (the saying period) was finished, the teacher began the assembly, or doing period. When the first part of the assembly was over, and she considered it an opportune moment to begin a psychomotor activity, she went over to the 5 experimental children (or called them together if they were not already sitting together) and applied the consequences for correspondence or lack thereof (see Table 1 above).

If one of the children had not stood up and had followed the teacher’s instructions, but had fought, then the teacher would socially reinforce the tasks the child had done correctly but would tell him that she could not give him the token.

She then asked the children to put the tokens in their uniform pocket and began to ask them questions (saying) relating to the next activity (the psychomotor function).

The procedure followed was similar to the other two situations (psychomotor function and worksheet activity). At the end of the morning, the children swapped their tokens for surprise envelopes the teacher had prepared beforehand. The intervention procedure was withdrawn partially from session 14 onwards. During this session, the teacher administered social consequences (approval, verbal feedback), but did not give the children tokens for two of the four activities that took place. The children were told that the teacher could not give them the tokens at that time, but that she would later on. In sessions 15 and 17, the surprise envelope element was also suppressed (the teacher told the children she had forgotten them). The consequences were not withdrawn totally because they were not disruptive, and neither was it necessary to do so; there are several references in the literature that support the use of these procedures with only intermittent use of consequences [56,60].

The interobservers’ reliability was calculated at 50% of the intervention sessions outside and inside the classroom using the formula [(Number of agreements/Number of agreements + disagreements) × 100]. For intervention outside the classroom, it was 100% for all 3 behaviors (B1, B2, and B3). For intervention inside the classroom conducted by the teacher, reliability was over 85% in all sessions, with an average of 91%.

### 2.5. Data-Analysis

To evaluate the effectiveness of the intervention, the average percentage of time per session each child spent behaving inappropriately (standing up + being absent from the task + being disruptive) by phase (baseline, intervention) was calculated. The non-parametric Wilcoxon test was then computed for related samples, comparing the baseline and intervention stages within each group. The non-parametric Mann-Whitney U Test for independent samples was calculated to compare the control group with the experimental groups during each stage.

## 3. Results

**Figure 1:**
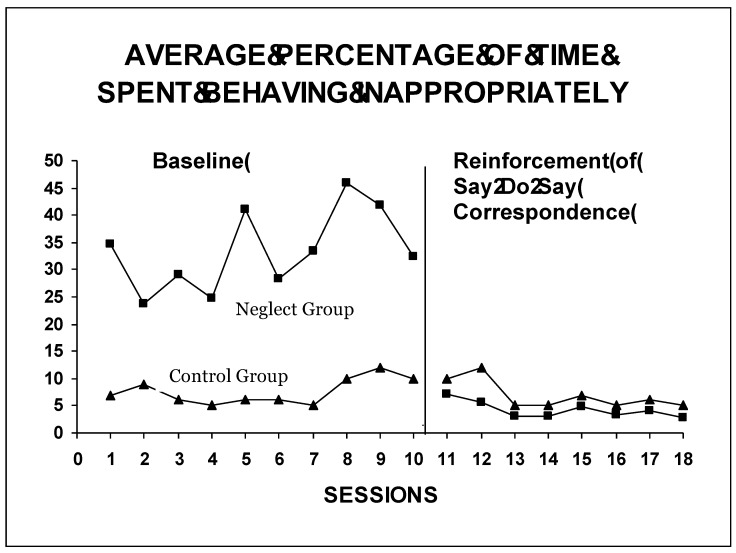
Graphic representation of the average percentage of time per session spent behaving inappropriately (standing up, absent from tasks, disruptive behaviors) during school activities, by the neglected group and the control group respectively. The x axis represents the sessions. Between sessions 10 and 11 took place the intervention outside the classroom carried out by the authors with the five neglected children.

The left side of the graph in Figure 1 shows the average percentages of time per session that the children in the control and the experimental groups spent behaving inappropriately (standing up + being absent from the task + being disruptive) during the pre-test or baseline period. These percentages varied: In the experimental group, between 23.75% and 46% of the total time the activity lasted was spent behaving inappropriately, whereas in the control group (non-neglected children) it was between 5% and 12%.

Intervention outside the classroom, conducted by the authors, took place between sessions 10 and 11 with the five neglected children in the experimental group. It consisted of training them in the S-D-S correspondence using the same procedure as that used by Herruzo and Pino [57]. The five children acquired the say-do correspondence for the trained behavior (B1) and then generalized it to the two new behaviors (B2 and B3). The process was completed with an average of 16.6 trials. The right part of Table 2 shows the results for each child.

Once the intervention outside the classroom was finished, intervention inside the classroom began with the experimental group, this time conducted by the teacher herself. This can be seen on the right of the graph in Figure 1, from session 11 onwards. The group was trained in the S-D-S correspondence: The children verbalized that they were not going to fight or stand up and that they were going to do whatever the teacher said, and they were encouraged when they kept their word, making them aware of the relationship between saying and doing. A drastic reduction was observed in the percentage of time spent behaving inappropriately, which fell to between 7.14% and 2.8%, whereas the children from the control group, where no intervention was carried out, maintained the levels shown in the pre-test or baseline stage.

The left part of Table 2 shows the mean amounts of time per session each child spent behaving inappropriately (standing up + being absent from the task + being disruptive) in the different stages (baseline, intervention).

The non-parametric Wilcoxon test on related samples revealed significant differences in the percentage of time spent behaving inappropriately (the dependent variable) between the baseline and the intervention stages (*p* = 0.043) within the intervention group, whereas none were found for the control group (*p* = 0.08). There were significant differences between the control group and the neglected group in the dependent variable during the baseline stage (Mann-Whitney U *p* = 0.008), but not during the intervention (Mann-Whitney U *p* = 0.151).

## 4. Discussion

The aim of this study was to improve classroom behaviors of neglected children using say-do-say correspondence training, firstly to learn say-do correspondence with a simple behavior outside of the classroom and generalize it to other, new, simple behaviors, and then with the teacher applying the correspondence training in the classroom to reduce out-of-task behaviors and improve in-task behaviors. After the intervention, a group of five neglected children who had originally spent a third of the time behaving inappropriately had this average percentage reduced to 4.5%, a normal level, equivalent to the percentage (7.6%) shown by the children in the control group (non-neglected children with whom no intervention was carried out).

The results of this study illustrate a promising new intervention procedure for children suffering from neglect only. They demonstrate the feasibility of the intervention and provided preliminary support for its potential benefits in reducing problematic classroom behaviors. They show that neglected children could improve their behavior in the classroom using say-do-say correspondence training. The study also confirms that in spite of the significantly delayed development these children displayed (associated with their situation of neglect), they can be treated and their behavior at school improved, with no need for any other change agent except their teacher. This means the intervention technique used can be classed as simple and easy to use. Above all, it is applicable over a short period of time and has very quick results. This is of great importance since techniques like this provide kindergarten teachers with an excellent tool to improve both the behavior of these children and their prognosis and because one of the major concerns for teachers is classroom management [23,38,60]. Nevertheless, as has been stated, this study constitutes only preliminary support. It must not be forgotten that it has several limitations (i.e., a very small sample size, non-randomization, generalizability), and thus further study, with rigorous research design such as randomized controlled trials to test the effectiveness of the intervention, is needed before this intervention can be recommended for use as an instrument to alleviate the negative effects of abuse.

On the other hand, the results obtained with the individual intervention used in this study replicate those obtained by Pino & Herruzo [57] and Ruiz et al. [49] in an applied context; this intervention successfully teaches neglected children self-control by training them in S-D-S correspondence, showing that this simple tool can be adapted in order to teach self-control to children who lack a repertoire in many areas. It is important to bear in mind that behaviors B1, B2, and B3, as has been demonstrated by Luciano et al. [54] and Herruzo and Pino [57], involved a self-control repertoire, since the subject has several toys in front of him and does not play with them, but does what he said he was going to; in other words, the child behaves in accordance with delayed consequences as opposed to other, more immediate ones. He manages to control his impulses.

The application of this procedure in groups, following individual intervention, achieved clearly visible results from the first session onwards, making it a useful and relatively unintrusive technique. The procedure is improved by a short intervention by the psychologist outside the classroom, preparing the child thus that the teacher can then modify his/her behavior more fundamentally. This obviates the tedious, costly stage of generalizing clinical interventions to the classroom. The authors believe that this technique is a promising instrument, suitable for children exposed to neglect and other types of maltreatment. The intervention can be used in schools and thus to alleviate the effects of abuse without the child having to be institutionalized. The teacher can carry on using the procedure to achieve other objectives in the classroom, and it could undoubtedly be adapted to specific objectives within a child’s own home, with or without parental collaboration, using the family educator as co-therapist. However, this is clearly beyond the scope of this study and will certainly be the subject of future research.

Another advantage of this intervention is the fact that the teacher’s perception of the pupils changed. As Little and Akin-Little [61] say, it is very important for classroom management procedures to improve teacher behavior, lowering teacher stress and enhancing student outcomes. The teacher spontaneously expressed a high level of satisfaction with the intervention because “it did not involve a lot of work and the children had improved a great deal”. She particularly highlighted subject S3, who went from being ‘absent’ for most of the time to being attentive all the time and participating more in activities. The child also began to complete the worksheets at the appropriate time, instead of standing up.

## 5. Conclusions

This article presents a promising new intervention procedure for reducing disruptive behaviors of neglected children in educational settings. Neglect is the most common, and the least researched type of maltreatment, and research into intervention techniques is particularly necessary. Since this technique can be easily implemented by teachers, it could be useful to professionals and paraprofessionals working with neglected children as a means of improving classroom behavior, helping children adapt better to school and, probably, also improving their future social adjustment. Nevertheless, this study provides preliminary support for the potential benefits of the intervention in reducing problematic classroom behaviors. Further study is needed before recommending that this intervention can be recommended for use as an instrument to alleviate the negative effects of abuse.

## Figures and Tables

**Table 1:** Training in the Say-Do-Say correspondence of several behaviors carried out by the teacher.

**Saying (Assembly)**
Teacher: “During the assembly, you must not stand up or fight, and you must do what I say we are all going to do”.
(Then she would ask one of the children)
Teacher: “Are you going to stand up?”
Child: “No”
Teacher: “Very good. Are you going to fight?”
Child: “No”
Teacher: “Very good. Are you going to do what I say? So if I say, put your hands up, what are you going to do?”
Child: “Put my hands up”
Teacher: “Very good, you’re not going to fight or stand up, and you’re going to do as I say. Very good. Now do not go and trick me, OK? “
Child: “OK”
Teacher: “If you do not trick me, I’ll give you a token”.
**Consequences for Correspondence (Assembly)**
Teacher: “I told you that during the assembly you must not stand up or fight and that you had to do as I said”
(Then she would ask the children one by one:)
Teacher: “Did you stand up?”
Child: “No”
Teacher: “And you?”
Child: “No”
Teacher: “Very good”. Until she had asked all five children. Then: “And did you fight?”
Child: “No”
Teacher: “And you?”
Child: “No”
Teacher: “Very good”. Until she has asked all five children. Then: “And did you do as I said?”
Child: Yes.
Teacher: “And you?”
Child: “Yes”
Teacher: “Very good”. (Until she had asked all five children:) Then she added: “so, did you trick me?”
Child: “No”
Teacher: “Do I have to give you the token?”
Child: “Yes”
Teacher: “Of course I do, because you did not trick me, here, have this token”. She then repeated this with the other children.
**If a Child does not Show Say-Do Correspondence**
Teacher: “I told you that during the assembly you must not stand up or fight and that you had to do as I said”
(Then she would ask the children one by one:)
Teacher: “Did you stand up?”
Child: “Yes”
Teacher: So, did you do what you said you were going to, or did you trick me?”
Child: “No” or “I tricked you”.
Teacher: “So, do I have to give you the token?”
Child: “No”
Teacher: “I am not going to give you the token because you tricked me. You said you were going to not stand up, but you did stand up. Because you did not do what you said you were going to, and you tricked me, I cannot give you this token. Let us try again next time”.

**Table 2:** Results specified by child.

SUBJECTS		Intervention Inside the Classroom		Intervention Outside the Classroom		
		Average Percentages of Time Spent Behaving Inappropriately		Number of Correct/Incorrect Trials of Say-Do Correspondence		Number of Trials to Reach the Criterion of Say-Do Correspondence
		(Baseline)	(Intervention)	Baseline	Intervention S-D-S	
**NEGLECT GROUP**	**S1**	39.6	9	0/9	5/8	13
	**S2**	19.7	2.1	0/9	2/7	9
	**S3**	31.2	3.87	0/9	13/27	40
	**S4**	44.7	4.1	0/9	2/7	9
	**S5**	31.9	3.71	0/9	3/9	12
**CONTROL GROUP**	**S6**	5.2	4.5			-
	**S7**	8	4.9			-
	**S8**	7.1	7.2			-
	**S9**	9.3	7.6			-
	**S10**	8.4	8.1			-

## Appendix A

**Table A1:** Neglect indices presented by neglected children (Group 1).

Age	Subject	Neglect Indices
4	S1	1,2,3,4,5,7
4	S2	1,2,5
4	S3	1,2,3,4,5,7
4	S4	2,3,4,5,6,7,8
4	S5	1,2,3,5,6,7

Indices:

1. Feeding: Adequate feeding has not been given to him or her. Hungry.
2. Clothing: The clothes are inadequate for the weather, badly kept from the cold.
3. Hygiene: Dirty all the time. Grimy. Insufficient hygiene.
4. Health care: Physical diseases without medical attention (e.g., infected injury or without care) or lack of control of usual medical needs.
5. Supervision: The child is frequently alone for a long time (or with another child) without adult surveillance.
6. Supervision: Accidents at home are usual due to the parent or care-taker neglect.
7. Safety and hygiene at home: Hygienic and/or physical condition of the home are dangerous to the child.
8. Educational area: Repeated and unjustified absence from school.
